# Zinc Protects against MDMA-Induced Apoptosis of Sertoli Cells in Mouse via Attenuation of Caspase-3 

**DOI:** 10.22074/ijfs.2020.44410

**Published:** 2020-10-12

**Authors:** Nadia Hossein-Zadeh, Morteza Bagheri, Isa Abdi Rad, Marziyeh Lozeie, Mahdieh Nasir-Zadeh

**Affiliations:** 1 Department of Genetic, Tabriz Branch, Islamic Azad University, Tabriz, Iran; 2 Cellular and Molecular Research Center, Cellular and Molecular Medicine Institute, Urmia University of Medical Sciences, Urmia, Iran

**Keywords:** Apoptosis, *Caspase-3*, MDMA, Sertoli Cells, Zinc

## Abstract

**Background:**

3,4-Methylenedioxymethamphetamine (MDMA) disrupts function of the endocrine system and dif- ferent organs such as heart, blood vessels, kidney, liver and nervous systems. This study was conducted to evaluate impact of MDMA on apoptosis and Zinc in the MDMA-induced apoptosis of cultured Sertoli cells by measuring* Caspase-3 *gene expression.

**Materials and Methods:**

In this experimental study, Sertoli cells were incubated with MDMA (0, 0.5, 1, 3, 5 mM), Zinc (0, 8, 16, 32, 64 μM) and Zinc (8 μM) prior to adding MDMA (5 mM) for 24 and 48 hours. MTT assay was used for evaluating impacts of these conditions on the viability of Sertoli cells. *Caspase-3* gene expression level was detected using quantitative reverse transcription PCR (qRT-PCR) in all of the tested groups.

**Results:**

Finding showed that cellular viability was decreased and level of Caspase-3 mRNA was increased in MDMA treated cells. Additionally, pre-treatment with Zinc (8 μM) attenuated MDMA-induced apoptosis and down-regulated caspase-3. The mean of caspase-3 mRNA level (fold change ± SE) was 3.98 ± 1.18, 0.31 ± 0.28, and 1.72 ± 0.28 in re- spectively MDMA (5 mM), Zinc (8 μM), and Zinc+MDMA groups vs. control group. The mean of *Caspase-3* mRNA (fold change) was not statistically different in the tested groups (P>0.05), unless MDMA (5 mM) group (P = 0.008).

**Conclusion:**

We suggest that MDMA toxicity could be involved in apoptosis of Sertoli cells. In addition, Zinc could reduce MDMA-induced apoptosis by down-regulation of *Caspase-3* mRNA levels.

## Introduction

3,4-Methylenedioxymethamphetamine (MDMA) as an illicit drug is used by young adults in the world (1). Since MDMA is consumed mainly by the young population, its effects on the reproductive system would be important to be taken into consideration (2,3). This hallucinogenic drug induces apoptotic cell death in different organs (4,5). MDMA affect the endocrine system and gonads (6). Prolonged usage of MDMA has several adverse effects on the pituitary-gonadal axis. This condition may affect the process of spermatogenesis (7). MDMA results in testicular edema and sperm DNA damage, low sperm count, immature/abnormal spermatozoa and asthenozoospermia. Poor semen quality can leads to male infertility (6). It has been demonstrated that numerous mechanisms are responsible for MDMA toxicity, including oxidative stress, metabolic compromise and inflammation (8). The deleterious impact of free radicals is termed oxidative stress causing serious biological damage (9). There is an excess of reactive oxygen species (ROS) and reactive nitrogen species (RNS) in biological systems regarding deficiency of antioxidants. The role of oxidative stress in MDMA toxicity has been observed by reducing the activity of endogenous antioxidants glutathione peroxidase, catalase (CAT) and superoxide dismutase (10). A Sertoli cell regulates spermatogenesis via controlling germ cell proliferation and apoptosis (11). Caspase-3 as a general activated death protease can catalyze the cleavage of cellular macromolecules such as DNA and proteins (12). Zinc as a fundamental trace element is essential to the structure and function of over 300 enzymes in endocrine system, immune system as well as anti-cancer defense mechanisms (13,14). Deficiency of Zinc leads to intrauterine growth retardation (delayed growth), appetite loss, pyrexia, cytokine storm and impaired reproductive capacity (15). Zinc get involved in NADPH oxidase generating ROS (16). Zinc induces production of metallothionein (MT) (17). Zinc deficiency has been associated with oxidative stress. In this regard, intracellular Zinc contents such as metallothionein regulate the oxidative stress. Zinc intake is associated with decreased level of ROS production in activated human neutrophils and decrease malone dialdehyde (MDA) (18). Here, we evaluated the effect of MDMA on apoptosis and Zinc on MDMA-induced apoptosis of cultured Sertoli cells by measuring *Caspase-3* gene expression.

## Materials and Methods

This experimental study was approved by research ethics committee of Urmia Medical Sciences University (Urmia, Iran; IR.UMSU.REC.1397.448). TM4 mouse cell line (Sertoli cells) was purchased from the Pasteur Institute of Iran and used at this study. TM4 cells were grown in DMEM/Nutrient Mixture F-12 Ham (Biosera, France), with 10% FBS (Gibco, USA) and 1% (v/v) Penicillin/ Streptomycin mixture (Gibco, USA). They were incubated at 37°C and 5% CO_2_ condition. The culture medium was replaced every 24 to 48 hours. The cultured cells were trypsinized after reaching 80% confluence by 0.25% trypsin-EDTA solution (Gibco, USA). Then, the cells were transmitted and cultured again on a new medium twice per week. The cells were cultured at a density of 5×10^3^ cells per well in 96-well culture plate (SPL, Korea). This was followed by incubation at 37°C and 5% CO_2_. After 24 -hours incubation, the cells were treated with MDMA (0, 0.5, 1, 3 and 5 mM; Sigma-Aldrich, USA) for 24 and 48 hours. Additionally, the cells were treated with 0, 8, 16, 32, 64 μM Zinc sulfate (Sigma, USA for 24 and 48 hours. After that, the supernatant was replaced with 100 μl PBS including 1 mg/ml MTT (3-(4, 5-Dimethylthiazol-2-yl)-2, 5-diphenyltetrazolium bromide; Sigma, USA). The samples were incubated at room temperature for 4 hours. Then, 100 μl dimethyl sulfoxide (Merck, Germany) was added and they were kept at room temperature for 15 minutes. Absorbance of each sample was measured at 590 nm with a microplate reader (Bio-Tek, USA). Data were reported as relative viability (% control).

Subsequently, the effective concentrations of MDMA and Zinc were determined. Then, TM4 cells were cultured in four groups and examined. They groups include MDMA, Zinc, Zinc pretreatment, and control group. These groups were subdivided in two subgroups regarding the 24 and 48 hours exposure time. The cells were cultured in 96-well plate at a density of 5×10^3^ cells / well, duplicately. The cells were twice cultured at the same time with identical density. After 48 hours exposure, the old medium was replaced by medium containing Zinc. After that time, the medium was replaced with MDMA-medium for the wells containing pre-treatment and MDMA groups, Zinc- medium for Zinc group and a free medium for control group. For the wells with 24 hours exposure, the same protocol 48 hours exposure period was applied, unless the starting pretreatment one day later. In addition, MDMA and Zinc exposure times were 24 hours. Zinc pre-treatment time was similar for both 24 and 48 hours subgroups, and only the treatment exposure time was different. MTT assay was then performed to evaluate cell viability of the different tested groups. To determine effective concentrations of MDMA and Zinc, these concentrations at 48 hours exposure time were used for the following experiments. In each set of triplicate cultures, 10^5 ^TM4 cells/wells were seeded in 6 well plates for 24 hours, followed by treatment consistent with the experimental main groups: MDMA (5 mM), Zinc (8 μM), pre-treatment and control (one well for each group). Following stabilization period of 24 hours, the well with pre-treatment group was exposed to Zinc and the other wells were exposed to free medium for the following 48 hours. The medium was replaced with a fresh medium in the midpoint of culture. The day after, the medium was aspirated from each well and replaced with MDMA containing medium for the wells regarding tested conditions. Forty eight hours after the last treatment, the cells were trypsinized and the cells pellet was used to extract RNA after centrifugation at 3000 rpm for 10 minutes. RNA extraction was carried out by the RNX Plus Solution Kit (SinaClon, Iran). In this study, forward and reverse primers for the target gene (*Caspase 3*) and reference gene (*β-actin*) were as follow:

*Caspase-3* (136 bp PCR product):

F: 5'-GCA GCT TTG TGT GTG TGATTC-3'

R: 5'-AGT TTC GGC TTT CCA GTC AG-3'

*β-actin* (250 bp PCR product):

F: 5'-TAG GCG GAC TGT TAC TGA GC-3'

R: 5'- GCT CCA ACC AAC TGC TGT C-3' (19).

Concentration of the RNA was confirmed for all samples and synthesis of cDNA with the same RNA concentration was performed by the following compounds: total RNA was used to produce cDNA using two-steps reverse transcription polymerase chain reaction (RTPCR) kit (Thermo Scientific, Sweden) as following: 10 μg RNA, 1 μl random Hexamers, 2 μl dNTPs mix (10 mM), 1 μl RiboLock RNase Inhibitor (20 U/μl), 4 μl 5X Reaction Buffer, 1 μl reverse transcriptase (RT) enzyme (200 U/μl). Final volume was reached to 20 μl, using nuclease-free water. The cDNA synthesis was carried out in a thermocyclor at 25°C for 5 minutes and 42°C for 60 minutes. Then, the synthesized cDNA was used to perform quantitative reverse transcription polymerase chain reaction (qRT-PCR). PCR program includes 95°C for 10 minutes (initial denaturation), 40 cycles of 95°C for 20 seconds (denaturation) and 60°C for 45 seconds (annealing). All samples were replicated two times. The relative expression level of Caspase-3 was normalized to β-actin. The results are reported based on Mean fold differences ± standard error (SE). All of the treated groups were compared to the control group. To analyze data, SPSS version 20 software was used. The statistically significant data was determined by one-way analysis of ANOVA and Tukey's test. To determine significance of the results, Threshold of P value was considered as 0.05. The qRT-PCR results were analyzed by the 2^-∆∆Ct^ method.

## Results

Cell viability and proliferation were determined using MTT assay. These were significantly reduced in the TM4 cells incubated with different concentrations of MDMA (0, 0.5, 1, 3, 5 mM) for 24 and 48 hours. In these two of exposure times, MDMA dose dependently decreased cell viability with IC50 values of 5 mM for 24 hours and 3 mM for 48 hours exposure (Fig . 1).

The cells were treated with various concentrations of Zinc (0, 8, 16, 32, 64 μM) for 24 and 48 hours. Cell viability was increased in the lower concentrations of Zinc in 24 and 48 hours exposure times, compared to the control Cell viability and proliferation were decreased as concentration of Zinc was increased (Fig . 2).

After analysis of the represented primary experiment data, 5 mM MDMA and 8 μM Zinc were considered as effective concentrations. The TM4 cells were exposed to 8 μM Zinc for 48 hours prior to adding MDMA. Cellular viability was then assessed and compared to the groups exposed to each of MDMA and Zinc. As shown in Figures 3 and 4, MDMA (5 mM) decreased cell viability, whereas cellular viability was increased in the Zinc (8 μM) group and those receiving Zinc before adding MDMA, in comparison with the control.

Amplifi cation efficiency was set at 90-105%. The results showed that the mean (± SE) of fold change was 3.98(± 1.18), 0.31(± 0.28), and 1.72(± 0.28) in MDMA (5 mM), Zinc (8 μM), and Zinc+MDMA groups, respectively. Mean of Caspase-3 mRNA expression level (fold change) was not statistically different in the tested groups (P value>0.05), unless in MDMA (5 mM) group (P value=0.008). In this regard, cellular viability and mean of Caspase-3 mRNA expression level (fold change) were similar in the control and Zinc+MDMA groups. These results implied that Zinc had a protective effect against MDMA induced-Sertoli cell apoptosis in mouse. Figure 5 shows a gel image of RT-PCR products in this study.

**Fig.1:**
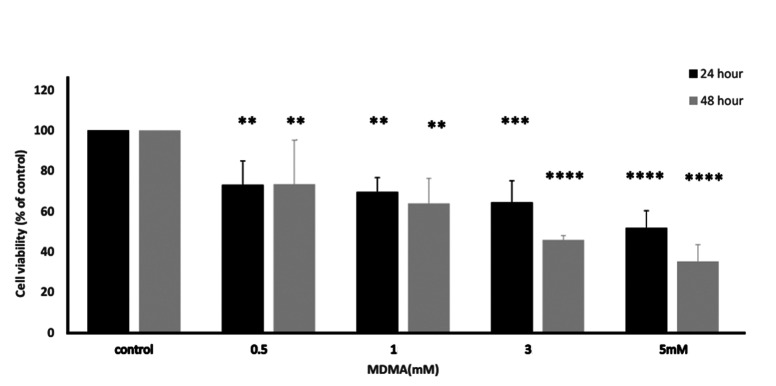
Impact of MDMA on TM4 cells. Evaluation of MDMA effect on TM4 cells in the tested groups showed significant differences in tested groups versus controls after 24 and 48 hours. **; P<0.01, ***; P<0.001, ****; P<0.0001, and MDMA; 3,4-Methylenedioxymethamphetamine.

**Fig.2:**
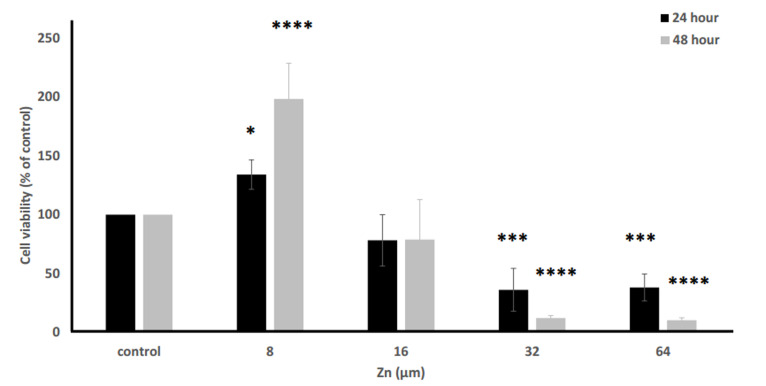
The impact of Zinc on TM4 cells. Analysis of the Zinc effect on the tested groups showed significant differences in the tested groups versus. controls after 24 and 48 hours. *; P<0.05, ***; P<0.01, and ****; P<0.001.

**Fig.3:**
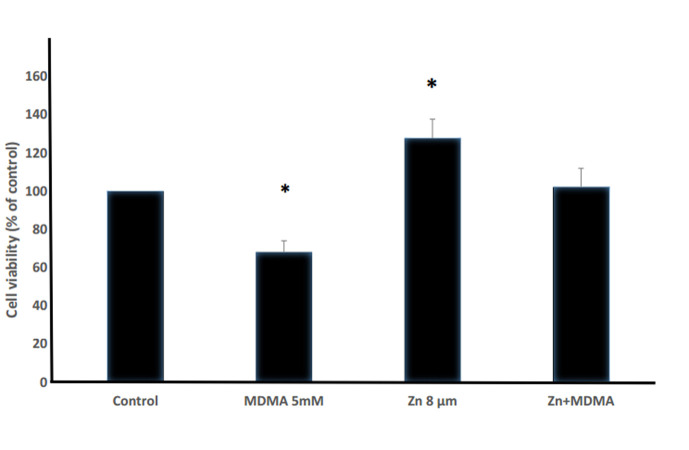
Comparison of cell viability in the tested groups versus control group. The experimental groups were treated with MDMA (5 mM) and/or Zinc(8 μM). In the Zinc+MDMA group, the cells were pre-treated with Zinc (8 μM) for 48 hours, before MDMA (5 mM) exposure. *; P<0.05, and MDMA; 3,4-Methylenedioxymethamphetamine.

**Fig.4:**
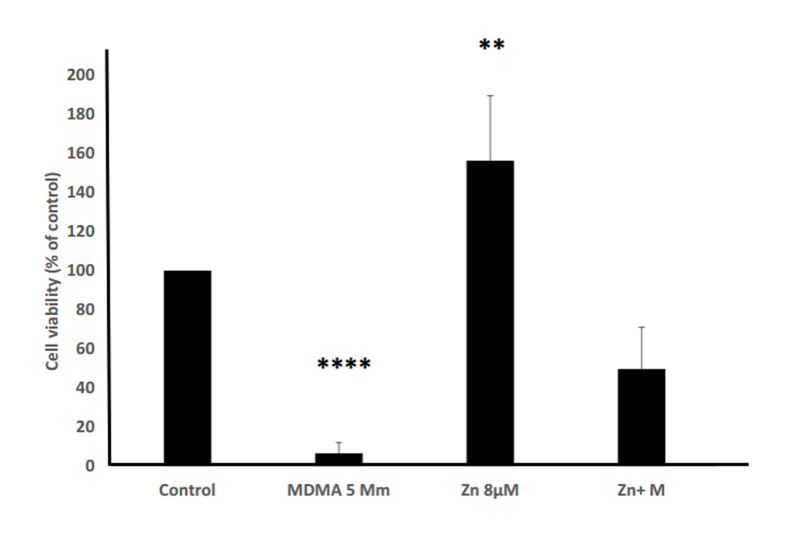
Comparison of the tested groups versus control group regarding cellular viability. The experimental groups were treated with MDMA (5 mM) and Zinc(8 μM). In the Zinc+MDMA group, the cells were pretreated with Zinc (8 μM) for 48 hours, before MDMA (5 mM) exposure. **; P<0.01, ****; P<0.001, and MDMA; 3,4-Methylenedioxymethamphetamine.

**Fig.5:**
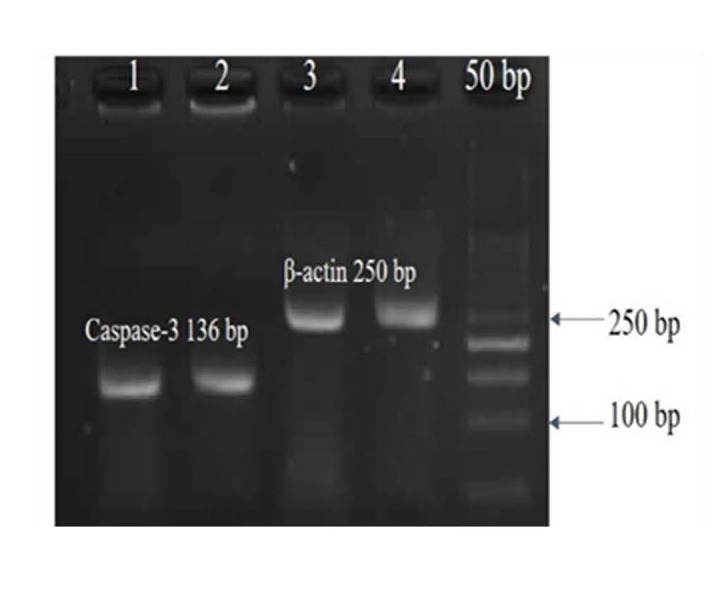
Reverse transcription polymerase chain reaction (RT-PCR) Analysis of tested genes by electrophoresis in a 2.5% agarose gel.

## Discussion

We investigated cell viability as well as *Caspase-3* mRNA expression level in the TM4 cells, incubated with MDMA, Zinc and Zinc+MDMA. We demonstrated that exposing cultured TM4 Sertoli cells to MDMA decreased cellular viability, while low concentration (8 μM) of Zinc reversed this condition. At high concentrations (16, 32 and 64 μM), Zinc had toxic effects and caused a high cellular death rate. Pre-treatment with Zinc, prior to MDMA exposure, attenuated MDMA-induced cellular toxicity. In addition, *Caspase-3* gene expression level was increased in MDMA-treated cells, while it was decreased in low concentration Zinc-treated cells. Pretreatment with Zinc decreased MDMA-induced elevation of *Caspase-3* mRNA expression levels. These findings showed that MDMA had cytotoxic and apoptotic effects on TM4 Sertoli cells, whereas Zinc (8 μM) had antiapoptotic effects and attenuated MDMA-induced cellular toxicity. It is well known that MDMA administration can induce oxidative stress (6, 8). It can harm the function of endocrine system and gonads (20). MDMA could influence male reproductive organs, cause sperm DNA damage. It also alters sperm count, sperm maturation and sperm mobility (6). MDMA induces cellular oxidative stress and apoptosis (21). While Sertoli cells have critical role in spermatogenesis (22), apoptosis of these cells leads to loss of germ cells. In the present study, our findings implied that MDMA had cytotoxic effect on Sertoli cells. It is well known that expression levels of caspase genes family is crucial in determining susceptibility of cells to apoptotic stimuli. *Caspase-3*, in particular, is a key player in this process and has many cellular targets, activation of which lead to apoptosis (23). Our study revealed MDMAinduced up-regulation and increase in *Caspase-3* mRNA expression level in the Sertoli cells. Decreased cellular viability and high level of *Caspase-3* gene expression in the MDMA treated-Sertoli cells may be defined as testicular injury that resulting in enhanced Sertoli cell apoptosis. It may be partly involved in infertility associated with MDMA consumption. Excessive ROS production and disrupting antioxidant defense of cells are associated with apoptotic effect of MDMA (24). In biological systems, the antioxidant properties of Zinc have been clearly demonstrated (25-27). Zinc supplementation induces metallothionein in diverse organs such as liver, kidney and intestine (28). Metallothionein has antioxidant effects (29). Zinc is known as a cofactor for Zn/Cu superoxide dismutase (SOD) enzyme, which acts as an ROS scavenger. This enzyme catalyzes alteration of O_2_ radical into the less harmful components of O_2_ and H_2_O_2_ (30). The protecting effect of Zinc against MDMA induced- Sertoli cell apoptosis seems to be related to its antioxidant properties. MDMA exerted cytotoxic property and induced-apoptosis through over-expression of *Caspase-3* in TM4 Sertoli cells. However, all of these parameters reversed or they recovered when the MDMA exposure was pretreated with Zinc. Only at 8 μM concentration of Zinc, we observed elevated cell viability after 24 and 48 hours exposure times. Maintaining right concentration of Zinc in the right physiological conditions can prevent oxidative stress in each cell of the body (30). In this study, high concentrations of Zinc had severe toxic effects on Sertoli cells and this finding is compatible to the previous studies.

## Conclusion

It can be concluded that Sertoli cells treatment with MDMA lead to decreased level of cell viability and induction of apoptosis by over-expression of *Caspase-3* mRNA level. Pre-treatment of Zinc, in advance to MDMA exposure attenuated MDMA-induced Sertoli cell apoptosis via inhibition of *Caspase-3* gene expression.
